# Prize Essays

**Published:** 1881-12

**Authors:** 


### PRIZE ESSAYS.

At the last meeting of the Medical Association of Georgia, that body, acting upon a suggestion contained in the address of the president, provided for the appointment of a committee on prize essays.

The committee was instructed to offer three prizes—one of fifty dollars, and two of twenty-five dollars each—for the best essays presented in accordance with the rules prescribed by the committee.

Acting under these instructions, the committee has addressed a circular letter to the physicians of the State, inviting them to compete for these prizes; and announcing the rules adopted by the committee in order to insure a fair and faithful execution of the trust.

In conformity with the resolution of the Association, and under the rules promulgated by the committee, the essays entered for the first prize must be on "Medical Plants, Natural and Foreign, Growing within the Limits of Georgia." A liberal range, in the discussion of the subject, is allowed, and the subdivisions of the question, proposed by the committee, are plainly set forth in the printed circular.

The second and third prize essays may be upon any subject, selected by the essayist, connected with medicine, surgery or hygiene.

It is not intended to limit the competition for these prizes to the members of the Association, but the contest is open to all physicians practicing within the State; all are invited to participate; and it is hoped by the committee that the invitation will meet with a generous response.

It was thought by the originators of this scheme that it would tend to arouse an increased interest in the State Association and serve to promote its prosperity and usefulness, besides, if it should prove successful, contributing largely to our knowledge of the therapeutic properties and uses of many indigenous and naturalized plants, at present unknown or but poorly understood.

The objects of this move are praiseworthy, and the efforts of the essayists, if wisely directed and conscientiously executed, will undoubtedly be productive of great practical good.

The prizes offered will be given in money or gold medals, suitably inscribed, of the full value of the respective amounts of the several prizes, at the option of the successful competitors.

We hope to see this effort for the promotion of medical organization within the State richly rewarded. It deserves success; we trust success may be achieved.

